# Six years of high-resolution climatic data collected along an elevation gradient in the Italian Alps

**DOI:** 10.1038/s41597-024-03580-x

**Published:** 2024-07-10

**Authors:** Alessandro Zandonai, Veronika Fontana, Johannes Klotz, Giacomo Bertoldi, Harald Crepaz, Ulrike Tappeiner, Georg Niedrist

**Affiliations:** 1 Institute for Alpine Environment, Eurac Research, viale Druso 1, 39100 Bolzano, Italy; 2 Department of Ecology, University of Innsbruck, Sternwartestr. 15, 6020 Innsbruck, Austria

**Keywords:** Climate and Earth system modelling, Atmospheric dynamics, Climate-change impacts, Climate-change mitigation, Projection and prediction

## Abstract

The complex meso- and microclimatic heterogeneity inherent to mountainous regions, driven by both topographic and biotic factors, and the lack of observations, poses significant challenges to using climate models to predict and understand impacts at various scales. We present here a six-year dataset (2017–2022) of continuous climatic measurements collected at five elevations from 983 m to 2705 m above sea level in the Val Mazia - Matschertal valley in the Italian Alps. The measurements include the air temperature, relative humidity, wind speed and direction, solar radiation, soil properties, precipitation, and snow height. Collected within the European Long-Term Ecological Research program (LTER), this dataset is freely available in an open access repository. The time series may be valuable for the validation of regional climate models, atmospheric exchange modelling, and providing support for hydrological models and remote sensing products in mountain environments. Additionally, our data may be useful for research on the influence of elevation on ecological processes such as vegetation growth, plant composition, and soil biology. Beyond its utility in advancing such fundamental research, meteorological monitoring data contribute to informed socio-political decisions on climate adaptation strategies, land management, and water resource planning, enhancing the safety and resilience of mountain communities and biodiversity.

## Background & Summary

Mountains are particularly sensitive to climate change, as several studies have shown^1–3^. This sensitivity extends to the speed of climate shifts^4^ and their impacts on both natural ecosystems^5^ and human societies^6^. However, climatic conditions in mountainous regions are subject to strong differences shaped not only by geomorphological factors such as the elevation, slope, or aspect^1^ but also by biotic factors such as the vegetation cover or soil type^7,8^. This high variability of climatic conditions makes it difficult to make general assumptions or to model and predict future changes^4^. Moreover, the systematic and long-term climatic monitoring of gradients within mountain ecosystems is rare^4,7^. Therefore, the fine-scale observation of key variables along elevation gradients is essential for obtaining a better understanding of micrometeorological and mesoclimatic variability over short distances^7^ and for assessing the impacts of climate change on mountain ecosystems using elevation as a climatic proxy^9^. With up to 20% of the world’s population living on mountains and their foothills^10^, long-term meteorological measurements along elevation gradients may contribute to anticipating the impacts of climate change in such sensitive regions.

Here, we present a dataset of continuous climatic measurements, collected at five different elevations over a distance of less than 15 km. The dataset includes the following measurements: the air temperature, relative humidity, wind speed and direction, solar radiation, soil temperature at 2, 5, and 20 cm depth, soil water content at 2, 5, and 20 cm depth, soil water potential at 5 and 20 cm depth, precipitation, and snow height. These data provide a detailed picture of meso- and microclimatic conditions along a mountain elevation gradient in an inner-alpine dry valley in the Italian Alps. The presented data were collected from 2017 to 2022 at the site Val Mazia – Matschertal within the framework of the European Long-Term Ecological Research (LTER) program, which studies ecosystems and their dynamics over time in all possible spheres, including the atmosphere and hydrosphere^11,12^.

Our six-year data set does not yet fulfil the temporal criteria to serve as a climate change reference, however it is of interest for applications on other levels. In the field of climate research, a local dataset from a mountainous region is useful for the bias correction of climate change simulations^13^ and the validation and downscaling of regional climate models and variables for complex terrain^14^. At a more local scale, observations along an elevation gradient are essential to better understanding, measuring, and modelling atmospheric exchange processes over mountains^15^.

The observations collected at the LTSER (Long-Term Socio-Ecological Research) site Val Mazia – Matschertal have already been proven useful for analysing the small-scale environmental variations of bio-physical variables and eco-hydrological processes associated with elevation^8^. In addition, this area serves as test site for validating catchment-scale hydrological models and processes^16–21^ or remote sensing products in mountain environments^22–25^. Furthermore, the dataset can contribute to addressing more specific research questions on the influence of elevation on ecological processes such as vegetation growth^26–28^, plant composition^29,30^, or biological soil processes^31,32^.

More broadly, and for example in combination with data collected at neighbouring LTER sites such as in Austria (e.g. Rofental^33^ or Obergurgl), our data allow for a detailed analysis of a meteorological profile across the main Alpine ridge and can provide relevant information for understanding the climate-induced impacts in the Earth’s Critical Zone^34,35^ of mountain regions. By supporting future land management or water resource planning, risks to mountain communities can be reduced and natural events such as landslides or avalanches, which are provoked by extreme weather conditions, can be better predicted^36^. This kind of action will make mountain communities and the surrounding biodiversity safer and more resilient.

## Methods

### Study area

**Fig. 1:**
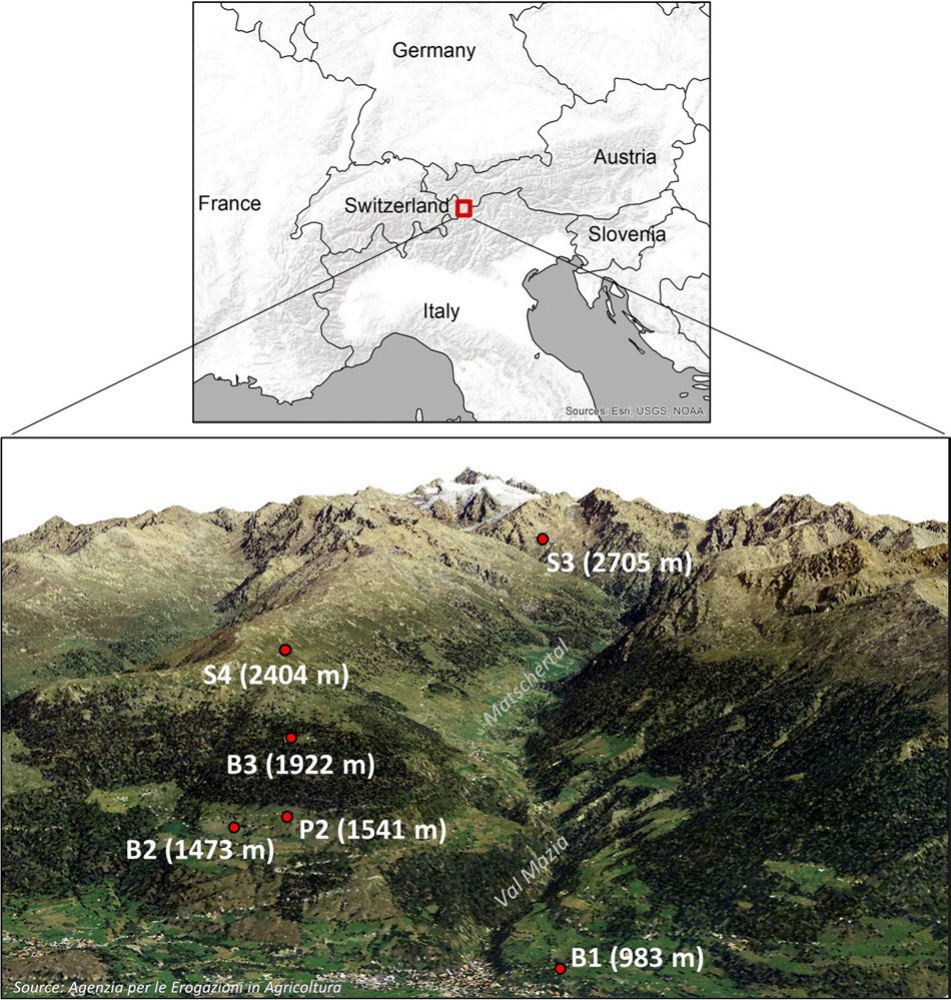
Overview of LTSER site Val Mazia – Matschertal located in the Italian Alps, with a visualisation of the six monitoring stations and the corresponding elevations.

The data were collected within the LTSER site Val Mazia – Matschertal (Fig. 1) (https://deims.org/11696de6-0ab9-4c94-a06b-7ce40f56c964), which is located in the province of Bolzano, South Tyrol, Italy (the northern boundary is located at latitude 46.766 N, the southern boundary at 46.661 N, the western boundary at longitude 10.585 E, and the eastern boundary at 10.710 E). Detailed information and an overview of the area covered by our LTER site can be found on the webpage: http://lter.eurac.edu/en.

The data series were recorded by six climate stations in the Mazia – Matschertal Valley where 24 climate stations are distributed over 90 km^2^. From this network of stations, we selected the five best-equipped stations in terms of the measurements presented, in order to be representative of an elevation transect of almost 2000 m (spanning from 983 m a.s.l. to 2705 m a.s.l.) (Fig. 1) from the lower mountain zone to the high alpine zone. One of the selected stations (P2, 1541 m a.s.l.) was not equipped with a net radiometer for solar radiation measurements and the rain gauge was installed only in July 2019. Hence, we included solar radiation measurements for the entire period and precipitation measurements from 2017 to July 2019 from a station 450 m away (B2, 1473 m a.s.l.). From July 2019 on, precipitation data originate from station P2. The Mazia – Matschertal Valley is characterised by inner-alpine continental climate conditions^37,38^. The average precipitation at 1922 m a.s.l. (climate station B3) is around 653 mm per year, with the maximum precipitation occurring in summer. The yearly average temperature is 4.6 °C (2017–2022).

### Local meso- and microclimatology

**Fig. 2:**
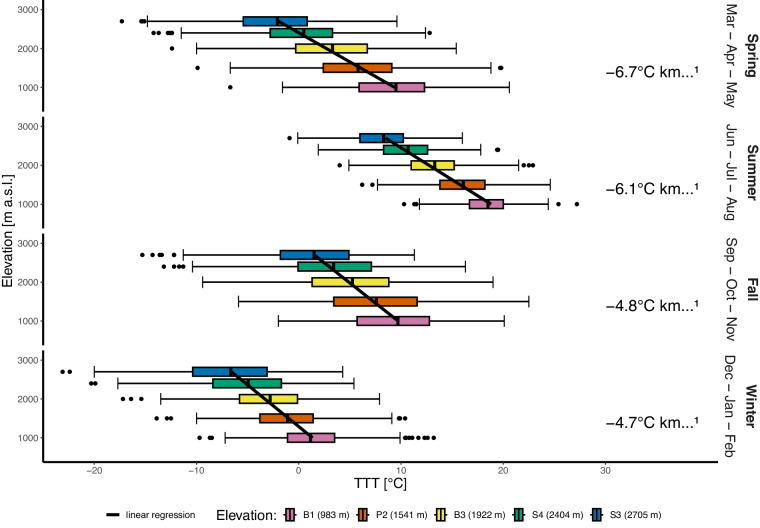
Differences in the variable air temperature (daily average) for the five monitoring stations along the elevational gradient, calculated separately for each of the four seasons. For each season, the temperature lapse rate has been calculated using linear regression. The box itself represents the interquartile range (IQR), which contains the middle 50% of the data. The bottom and top edges of the box correspond to the first quartile (Q1) and the third quartile (Q3), respectively. The line inside the box indicates the median (Q2) of the data. The whiskers extend from the edges of the box to the smallest and largest values within 1.5 * IQR from the first and third quartiles. The points represent outliers, defined as values that are below Q1 - 1.5 * IQR or above Q3 + 1.5 * IQR.

We present here some examples of the potential use of the data to describe the local climatology along a mountain elevation transect. The seasonal air temperature lapse rate (Fig. 2) ranges from −6.7 °C to −4.7 °C per 1000 m of elevation in spring and winter, respectively. Data show a lower lapse rate in winter, which is related to the frequent thermal inversion conditions in the valley bottom.

**Fig. 3:**
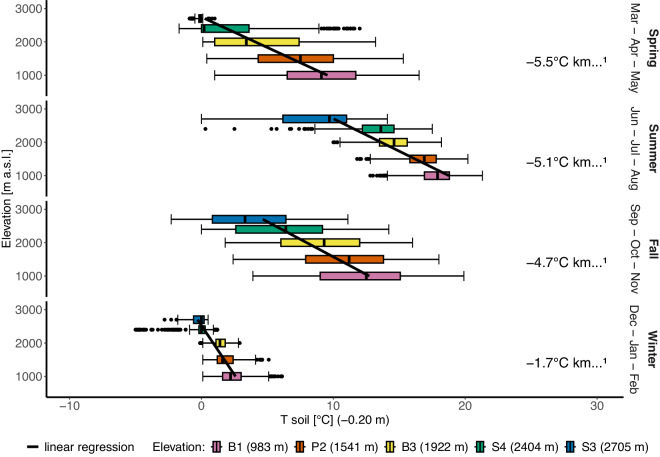
Differences in the variable soil temperature at 20 cm depth (daily average) for the five monitoring stations along the elevational gradient, calculated separately for each of the four seasons. For each season, the temperature lapse rate has been calculated using linear regression. The box itself represents the interquartile range (IQR), which contains the middle 50% of the data. The bottom and top edges of the box correspond to the first quartile (Q1) and the third quartile (Q3), respectively. The line inside the box indicates the median (Q2) of the data. The whiskers extend from the edges of the box to the smallest and largest values within 1.5 * IQR from the first and third quartiles. The points represent outliers, defined as values that are below Q1 - 1.5 * IQR or above Q3 + 1.5 * IQR.

The same visualisation can be made with a less frequently measured variable, such as the soil temperature at 20 cm depth, which is a key element in hydrothermal processes at the land-atmosphere boundary, linking the surface structure to physical and biological soil processes^37^. Figure 3 shows that the soil temperature lapse rate is lower than the air temperature lapse rate, ranging from −5.5 °C to −1.7 °C per 1000 m of elevation. During the winter season, there is less variability in the soil temperature because of the insulating effect of the snow and the energy exchange of soil freezing and melting cycles. Only at stations above 2000 m a.s.l. is the soil permanently frozen in winter. The coldest temperatures were registered at station S4 (2400 m) during a phase of low snow cover and not, as expected, at the higher station S3 (2700 m). In summer and especially in spring, however, the highest station, S3, is much colder than the other stations. The very low soil temperature variability at S3 in spring can be explained by the thick snow cover that is still present at 2700 m.

**Fig. 4:**
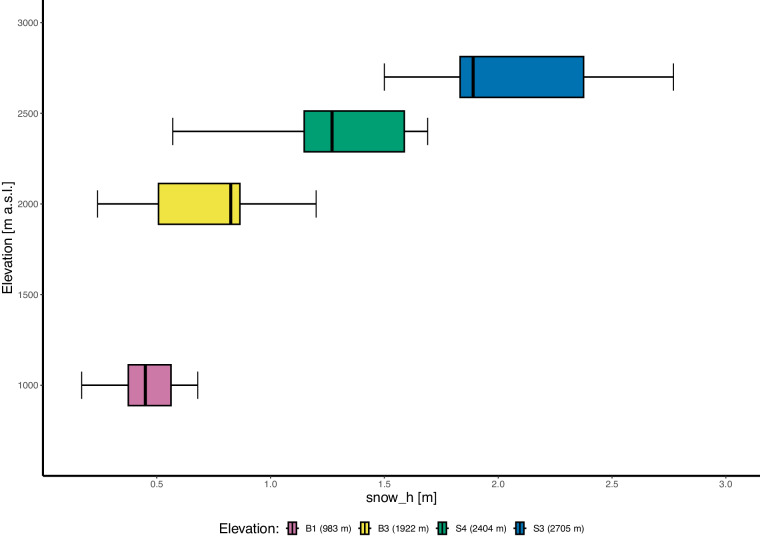
Snow height at the elevation of each station, calculated as a yearly maximum for the period when snow is present. Snow height was not recorded at station P2 (1541 m a.s.l.). The box itself represents the interquartile range (IQR), which contains the middle 50% of the data. The bottom and top edges of the box correspond to the first quartile (Q1) and the third quartile (Q3), respectively. The line inside the box indicates the median (Q2) of the data. The whiskers extend from the edges of the box to the smallest and largest values within 1.5 * IQR from the first and third quartiles.

Indeed, the valley shows a strong elevational gradient in the snow height, which is not linear with elevation. The average maximum snow height during the winter season ranges from 30 cm at 1000 m a.s.l. to more than 2 m at the highest station at 2700 m a.s.l. (Fig. 4).

### Monitoring stations

**Fig. 5:**
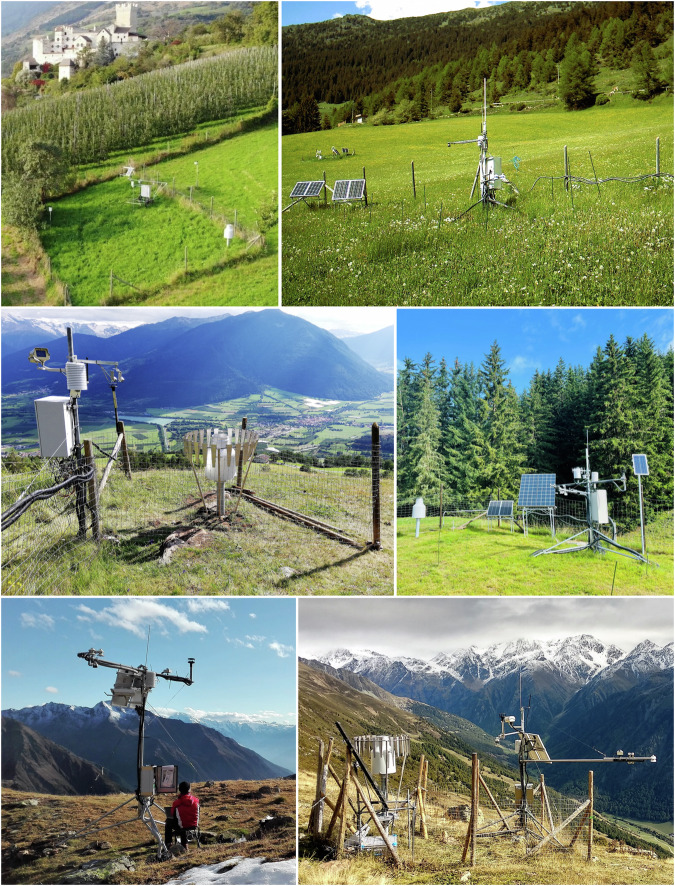
Pictures displaying the monitoring stations and the surrounding landscape. Upper left: station B1 (983 m a.s.l.). Upper right: station B2 (1473 m a.s.l.). Center left: station P2 (1541 m a.s.l.). Center right: station B3 (1922 m a.s.l.) Lower left: station S3 (2705 m a.s.l.). Lower right: station S4 (2404 m a.s.l.).

The six monitoring stations consist of steel structures, which are usually protected by a wooden fence, and an electrified fence to prevent wild and domestic animals from entering the measurement area and damaging structures and equipment (Fig. 5). A box is mounted on the structure that houses the devices necessary for data acquisition and transmission, as well as the power supply for the various components, which obtain power via a 12 V photovoltaic (PV) system. Topographical details, land cover and soil properties around each monitoring station are given in Table 1.

**Table 1:** Description of the monitoring stations that includes the geographic coordinates, elevation, terrain exposure, slope, surrounding land use, and soil composition.

Station	Latitude [DD]	Longitude [DD]	Elevation a.s.l. [m]	Slope [deg]	Aspect [deg]	Land Use	Soil Type	Texture	Soil Depth [m]	Sand [%]	Silt [%]	Clay [%]	Humus [cm]
B1	46.661183	10.590244	983	12	230	Irrigated meadow	Brown earth	Sandy loam	0.80	56.2	37.7	6.1	6.1
B2	46.686253	10.579917	1473	14	220	Irrigated Meadow	Brown earth	Sandy loam	0.66	52.7	38.6	8.7	14.4
P2	46.684306	10.585125	1541	27	230	Pasture	Brown earth/Ranker	Loam	0.44	44.0	42.0	14.0	11.5
B3	46.691694	10.591936	1922	11	220	Pasture	Ranker	Loam	0.64	48.9	39.4	11.7	18.9
S4	46.706981	10.602181	2404	22	135	Pasture	Ranker	Loam	0.24	51.1	30.3	18.6	9.1
S3	46.766786	10.710939	2705	14	230	Pasture	Ranker	/	0.14	/	/	/	23.0

The acquisition system consists of a logger (Campbell Scientific CR1000/CR1000X) and an expansion module for the logger inputs (Campbell Scientific multiplexer AM16/32B). The box also contains a 2 G modem or a 4 G router equipped with a machine-to-machine (M2M) SIM card for the transmission of data from the logger to the file server and, more generally, for the remote monitoring and maintenance of the stations. Thanks to a dynamic domain name server (DDNS) service, it is also possible to remotely access and connect to the stations. The modem/router is linked to an external omni-directional antenna, which can also be directional (Yagi antenna) for more remote stations where the mobile phone signal is particularly poor.

A 12 V/40 Ah battery is usually placed inside the box and connected to a 5 A charge regulator, which is in turn connected to one or two 30 Wp PV panels mounted outside: this dimensioning, supported by careful in-house consumption management, guarantees autonomy of approximately one week in the event of prolonged bad weather.

At some of the stations located at high elevations, specifically B3 (1922 m a.s.l.) and S4 (2404 m a.s.l.), the rain gauges have been equipped with orifice rim heating. Each of these stations also has a 24 V PV power supply system with a 10 A regulator and 4 × 100 Ah batteries housed in a dedicated box; this system is completed by a pair of 140 Wp PV panels. Table 2 describes in detail the equipment of the stations in terms of the data acquisition system (excluding sensors), the transmission system, and the PV power supply systems.

**Table 2:** Current station equipment, including the data acquisition system, data transmission system, and photovoltaic power supply system.

EQUIPMENT DESCRIPTION				MEASUREMENT STATION (Device presence and quantity)					
System	Device	Brand	Type/Model	B1 983 m	B2 1473 m	P2 1541 m	B3 1922 m	S4 2404 m	S3 2705 m
Data acquisition	Logger	Campbell Scientific	CR1000	1	1	1	1	1	
		Campbell Scientific	CR1000X						1
	Logger O.S.	Campbell Scientific	Version	32.03	32.05	32.05	32.04	32.05	6.0
	Multiplexer	Campbell Scientific	AM16/32B	1	1		1		
Data transmission (mobile network)	Modem and router	Teltonika	Router 4 G RUT‐240		1	1	1	1	1
		Telit	Modem 2 G GT863‐PY	1					
	Antenna	Siretta	Omnidirectional ‐ Oscar 1 – 4 dBi		1	1		1	
		Campbell Scientific	Omnidirectional ‐ Dual‐band	1					
		RF Solutions	Directional ‐ YAGI for GSM – 11 dBi						1
		Poynting	Directional ‐ LPDA‐92 – 12 dBi				1		
Photovoltaic power supply 12 V (acquisition and transmission)	Panel	NX Solar	NX30P – 30 Wp	1		1	2	2	2
		HighLine	SME 50 – 50 Wp		2				
	Battery	Prime	AGM ‐ PCA38‐12 – 40 Ah			1			1
		Haze	GEL ‐ HZY‐EV12‐44 – 45 Ah				1	1	
		Viktron	GEL – 60 Ah	1					
		Haze	AGM - HZB12-100 - 115Ah		1				
	Charger	EPsolar	LandStar (PWM) 12 V/5 A	1	1	1	1	1	1
Photovoltaic power supply 24 V (rain gauge heating)	Panel	Kyocera	KD140GH‐2YU – 140 Wp				2		
		Kyocera	KD140GH‐2PU – 140 Wp					2	
	Battery	Prime	AGM ‐ PCA100‐12 – 100 Ah				4	4	
	Charger	Steca	Solarix MPPT 1010 – 24 V/10 A				1		
		Epever	Tracer 2210 A (MPPT) – 24 V/10 A					1	

### Sensor equipment and variable collection

Analogue and digital sensors, as well as single and multi-parametric sensors, were used to collect the 15 variables (Table 3). In general, the dataset values are the result of averaging 15 records taken every minute and aggregating them into a single value stored in the logger, which is then transferred to our internal file server. However, there are exceptions to this process: precipitation is recorded as the total amount of precipitation, and for the snow height, only the last of the 15 records is stored to minimise the impact of outliers.

**Table 3:** Description of the measurements, including information on which variables are collected at each station, variable abbreviations and units.

MEASUREMENT				MEASUREMENT STATION					
Name	Abbreviation	Unit	Sample Aggregation*	B1 983 m	B2^^^ 1473 m	P2 1541 m	B3 1922 m	S4 2404 m	S3 2705 m
Temperature, air	TTT	°C	Average	X		X	X	X	X
Humidity, relative	RH	%	Average/sample**	X		X	X	X	X
Wind speed	ff	m/s	Average	X		X	X	X	X
Wind direction	dd	deg	Average/sample***	X		X	X	X	X
Short-wave downward (GLOBAL) radiation	SWD	W/m^2^	Average		X		X	X	X
Precipitation	Precip	mm	Total	X	X	X	X	X	
Snow height	Snow h	m	Average/sample****	X			X	X	X
Temperature, soil (−0.02 m)	T soil (−0.02 m)	°C	Average	X		X	X	X	X
Temperature, soil (−0.05 m)	T soil (−0.05 m)	°C	Average	X		X	X	X	X
Temperature, soil (−0.20 m)	T soil (−0.20 m)	°C	Average	X		X	X	X	X
Soil water content, volumetric (−0.02 m)	vol SWC (−0.02 m)	m^3^/m^3^	Average	X		X	X	X	X
Soil water content, volumetric (−0.05 m)	vol SWC (−0.05 m)	m^3^/m^3^	Average	X		X	X	X	X
Soil water content, volumetric (−0.20 m)	vol SWC (−0.20 m)	m^3^/m^3^	Average	X		X	X	X	X
Soil water potential (−0.05 m)	Psi Soil (−0.05 m)	kPa	Average	X		X	X	X	X
Soil water potential (−0.20 m)	Psi Soil (−0.20 m)	kPa	Average	X		X	X	X	X

* Aggregation method:	Sample	The last of 15 samples is logged
	Average	The average of 15 samples is logged
	Totalize	The sum of 15 samples is logged

Exceptions^^	**Station**	**Sample Aggregation**	**Average Aggregation**
**RH	*P2*	*Until 2017‐06‐01 09:45*	*from 2017-06-01 10:00*
	*B3*	*Until 2017‐06‐13 10:45*	*from 2017-06-13 11:00*
***dd	*B1*	*Until 2021‐08‐25 16:00*	*from 2021-08-25 16:15*
	*P2*	*Until 2021‐03‐19 18:30*	*from 2021-03-19 18:45*
	*B3*	*From 2017‐06‐15 16:15 to 2021‐08‐05 12:45*	*until 2017-06-15 16:00 & from 2021-08-05 13:00*
	*S4*	*Until 2021‐08‐06 17:45*	*from 2021-08-06 18:00*
	*S3*	*Until 2021‐08‐07 09:45*	*from 2021-08-07 10:00*
****Snow h	*S4*	*from 2017-09-26 06:45*	*Until 2017‐09‐26 06:30*
	*S3*	*from 2017-03-08 10:45*	*Until 2017‐03‐08 10:30*

Information on sample aggregation is provided for each measurement. We adopted the variable names, the corresponding abbreviations, and the units of measurement used by the Pangaea repository.

^*^ Sampling period: 1 min; Aggregation and logging period: 15 min. ^^Exceptions: measurements aggregated with different methods in different periods.

^ Complementary station for P2 (installed 450 meters away).

Although we have tried to keep the measurement setups as similar as possible, there are some differences between the monitoring stations (indicated in Table 3).

An overview of the sensors is given in Table 4, where their model and brand and the measured variables, including the measurement range and accuracy, are listed.

**Table 4:** Description of the sensors used for each measurement, including the brand, sensor model, range, and accuracy according to the manufacturer.

Sensor	Brand	Model	Measured variable	Range	Accuracy
Thermo-hygrometer and solar radiation shield	Rotronic	HC2S3	Temperature, air Humidity, relative	−40 to 60 °C 0 to 100%	±0.1 °C at 23 °C ±0.8% at 23 °C
	Vaisala	HMP45C	Temperature, air Humidity, relative	−39.2 to 60 °C 0.8 to 100%	±0.2 °C at 20 °C ±2.0% at 20 °C (0–90%)
	Vaisala	HMP155A	Temperature, air Humidity, relative	−80 to 60 °C 0 to 100%	±0.1 °C at 20 °C ±1.0% (15 to 25 °C) (0–90%)
2D anemometer	R. M. Young	Wind Sentry Set 03002	Wind speed Wind direction	0 to 50 m/s 0 to 359°	±0.5 m/s ±5°
	Gill	WindSonic1	Wind speed Wind direction	0 to 60 m/s 0 to 359°	±0.24 m/s at 12 m/s ±3° at 12 m/s
	Gill	WindSonic4	Wind speed Wind direction	0 to 60 m/s 0 to 359°	±0.24 m/s at 12 m/s ±3° at 12 m/s
Rain gauge (weight-based)	Ott	Pluvio2 (400 cm^2^)	Precipitation	0.05 to 500 mm/h	±0.1 mm (−25 to 45 °C)
Ultrasonic distance sensor	Campbell Scientific	SR50A	Snow height	0.5 to 10 m	±0.01 m or 0.4%
	Campbell Scientific	SR50AT	Snow height Temperature, air	0.5 to 10 m −45 to 50 °C	±0.01 m or 0.4% ±0.2 °C < 0 °C,±0.75 °C > 0 °C
Thermopile pyranometer (Four-component net radiometer)	Hukseflux	SR01 (NR01)	Short-wave downward (GLOBAL) radiation	305 to 2800 nm 0 to 2000 W/m^2^	±10% for daily totals
	Apogee	SP-510 (SN-500)	Short-wave downward (GLOBAL) radiation	385 to 2105 nm 0 to 2000 W/m^2^	±5% for daily totals
Soil water content reflectometer	Campbell Scientific	CS655	Soil water content, volumetric Temperature, soil	0 to 52% −10° to + 70 °C	±3% (EC ≤ 10 dS/m) ±0.5 °C
Dielectric water potential sensors	Decagon	MPS-6	Soil water potential Temperature, soil	−9 to −10^5^ kPa −40° to + 60 °C	±10% + 2 kPa (−9 to −100 kPa)±1.0 °C
	Meter Group	Teros 21	Soil water potential Temperature, soil	−9 to −10^5^ kPa −40° to + 60 °C	±10% + 2 kPa (−9 to −100 kPa)±1.0 °C

Throughout the six years of data collection, data gaps due to sensor failure (including data loss due to maintenance interventions) amounted to 2% of the dataset. Various circumstances have required the replacement of singular sensors or more involved maintenance work. We highlight two of those cases, one due to anomalies being detected and the other due to data quality improvement.

In the case of temperature and humidity measurements, for which we initially relied on Rotronic mod. HC2S3 thermo-hygrometers, we noticed that after a few years of operation, anomalous peaks had appeared in the temperature measurements of all the installed sensors. After a more detailed analysis of the data series, we found that in addition to these peaks, the measurements taken over the following weeks were also noisy and unreliable. After replacing these sensors with the Vaisala HMP155 model, we no longer experienced this anomaly.

With the aim of improving the data quality of the variable precipitation, we upgraded the weight-based rain gauges (mod. Pluvio2, made by Ott) at two stations, B3 and S4, which are located at elevations of 1922 and 2404 m a.s.l., respectively. The heating of the orifice rim of the rain gauge bucket was added in order to minimise the underestimation of solid precipitation, which in certain cases can remain attached to the rim. In addition, we improved the stations’ own data acquisition scripts to allow them to identify solid precipitation events with high accuracy, using an approach developed by Mair *et al*.^39^. This approach automatically activates the heater on demand, optimising energy consumption and ensuring that the heater operates throughout the winter. To improve the performance of the rain gauges, wind shields were also installed at the windiest stations, which are P2 (1541 m a.s.l.) and S4.

The complete sensor history for each monitoring station can be found in Tables 5–10, which also include the operating periods and the upgrades made to each station.

**Table 5:** Sensor history for monitoring station B1 (983 m), describing sensor replacements and upgrades from 2017 to 2022.

Station B1 (983 m)				
Measured variable	Sensor model/setup	Sensor height [m]	Period of operation	
			Start	End
Temperature, air - Humidity, relative	HMP45C	1.80	2009-11-26	2019-07-08
	HC2S3	1.80	2019-08-06	2019-08-22
	HMP155A	1.90	2019-08-22	ongoing
Wind speed - Wind direction	Windsonic1	2.00	2009-11-26	2019-08-06
	Windsonic4	2.00	2019-08-06	ongoing
Precipitation	Pluvio2	1.50	2015-12-10	ongoing
Snow height	SR50AT	1.90	2009-11-26	ongoing
Soil water content, volumetric - Temperature, soil	CS655	−0.02	2016-04-14	ongoing
	CS655	−0.05	2016-04-14	ongoing
	CS655	−0.20	2016-04-14	ongoing
Soil water potential	MPS-6	−0.05	2016-04-20	2017-12-19
	MPS-6	−0.05	2017-12-19	ongoing
	MPS-6	−0.20	2016-04-20	ongoing

**Table 6:** Sensor history for monitoring station B2 (1473 m), describing sensor replacements and upgrades from 2017 to 2022.

Station B2 (1473 m)				
Measured variable	Sensor model/setup	Sensor height [m]	Period of operation	
			Start	End
Short-wave downward (GLOBAL) radiation	NR01	1.20	2010-10-14	ongoing
Precipitation	Pluvio2	1.50	2015-12-10	2017-08-30
	Pluvio2 (ex. S4 station)	1.50	2017-08-30	2019-07-09
	(Wind shield added)	1.50	2018-11-06	2019-07-09

**Table 7:** Sensor history for monitoring station P2 (1541 m), describing sensor replacements and upgrades from 2017 to 2022.

Station P2 (1541 m)				
Measured variable	Sensor model/setup	Sensor height [m]	Period of operation	
			Start	End
Temperature, air - Humidity, relative	HC2S3	2.00	2016-06-26	2018-11-21
	HC2S3	2.00	2018-11-21	2019-08-22
	HMP155A	2.00	2019-08-22	2019-10-08
	HMP155A	2.00	2019-10-08	ongoing
Wind speed - Wind direction	Wind Sentry Set 03002	2.00	2014-04-09	2017-06-01
	Windsonic4	2.00	2017-06-01	ongoing
Precipitation	Pluvio2 + wind shield (ex B2 station)	1.50	2019-07-09	ongoing
Soil water content, volumetric - Temperature, soil	CS655*	−0.02	2014-06-16	ongoing
	CS655*	−0.05	2014-04-09	ongoing
	CS655*	−0.20	2014-04-09	ongoing
Soil water potential	MPS-6	−0.05	2019-03-20	ongoing
	MPS-6	−0.20	2019-03-20	ongoing

* Sensor installed in the area “Plot A” of the monitoring station P2.

**Table 8:** Sensor history for monitoring station B3 (1922 m), describing sensor replacements and upgrades from 2017 to 2022.

Station B3 (1922 m)				
Measured variable	Sensor model/setup	Sensor height [m]	Period of operation	
			Start	End
Temperature, air - Humidity, relative	HMP45C	2.00	2009-11-25	2018-05-08
	HC2S3	2.00	2018-05-08	2019-06-13
	HC2S3	2.00	2019-06-13	2019-06-28
	HC2S3	2.00	2019-06-28	2019-08-22
	HMP155A	2.00	2019-08-22	ongoing
Wind speed - Wind direction	Windsonic1	2.00	2009-11-25	2017-06-13
	Windsonic4	1.75	2017-06-13	ongoing
Short-wave downward (GLOBAL) radiation	SN-500	1.50	2019-06-13	ongoing
Precipitation	Pluvio2	1.50	2015-10-20	ongoing
	(Heater added)	none	2019-10-30	ongoing
Snow height	SR50AT	1.75	2009-11-25	ongoing
	CS655	−0.02	2015-10-20	ongoing
Soil water content, volumetric - Temperature, soil	CS655	−0.05	2015-10-20	ongoing
	CS655	−0.20	2015-10-20	ongoing
Soil water potential	MPS-6	−0.05	2015-10-20	ongoing
	MPS-6	−0.20	2015-10-20	ongoing

**Table 9:** Sensor history for monitoring station S4 (2404 m), describing sensor replacements and upgrades from 2017 to 2022.

Station S4 (2404 m)				
Measured variable	Sensor model/setup	Sensor height [m]	Period of operation	
			Start	End
Temperature, air - Humidity, relative	HC2S3	2.00	2016-09-29	2018-11-22
	HC2S3	2.00	2018-11-22	2019-08-23
	HMP155A	2.00	2019-08-23	ongoing
Wind speed - Wind direction	Windsonic4	2.65	2016-09-29	ongoing
Short-wave downward (GLOBAL) radiation	NR01	2.20	2016-09-29	ongoing
Precipitation	Pluvio2 + wind shield	1.50	2016-09-29	2017-08-30
	Pluvio2 (ex B2 station) + wind shield	1.50	2017-08-30	ongoing
	(Heater added)	none	2017-09-26	2022-06-01
	(Pluvio & wind shield lifted up)	2.00	2018-11-22	ongoing
Snow height	SR50A	2.15	2016-09-29	2022-05-31
	SR50A	2.15	2022-05-31	ongoing
Soil water content, volumetric - Temperature, soil	CS655	-0.02	2016-09-29	ongoing
	CS655	-0.05	2016-09-29	ongoing
	CS655	-0.20	2016-09-29	ongoing
Soil water potential	MPS-6	-0.05	2016-10-27	ongoing
	MPS-6	-0.20	2016-10-27	2018-11-29
	Teros 21	-0.20	2018-11-29	ongoing

**Table 10:** Sensor history for monitoring station S3 (2705 m), describing sensor replacements and upgrades from 2017 to 2022.

Station S3 (2705 m)				
Measured variable	Sensor model/setup	Sensor height [m]	Period of operation	
			Start	End
Temperature, air - Humidity, relative	Rotronic HC2S3	3.00	2016-10-31	2018-08-03
	Rotronic HC2S3	3.00	2018-08-03	2019-09-18
	Vaisala HMP45C	3.00	2019-07-08	2019-09-18
	Vaisala HMP155A	2.70	2019-09-17	ongoing
Wind speed - Wind direction	Windsonic4	3.30	2016-09-14	ongoing
Short-wave downward (GLOBAL) radiation	NR01	3.10	2016-09-14	ongoing
Snow height	SR50A	3.05	2016-10-31	2020-07-02
	(SR50A lifted down)	2.80	2020-07-02	ongoing
Soil water content, volumetric - Temperature, soil	CS655	-0.02	2016-09-14	ongoing
	CS655	-0.05	2016-09-14	ongoing
	CS655	-0.20	2016-09-14	ongoing
Soil water potential	MPS-6	-0.05	2016-09-14	ongoing
	MPS-6	-0.20	2016-09-14	ongoing

### Data workflow

**Fig. 6:**
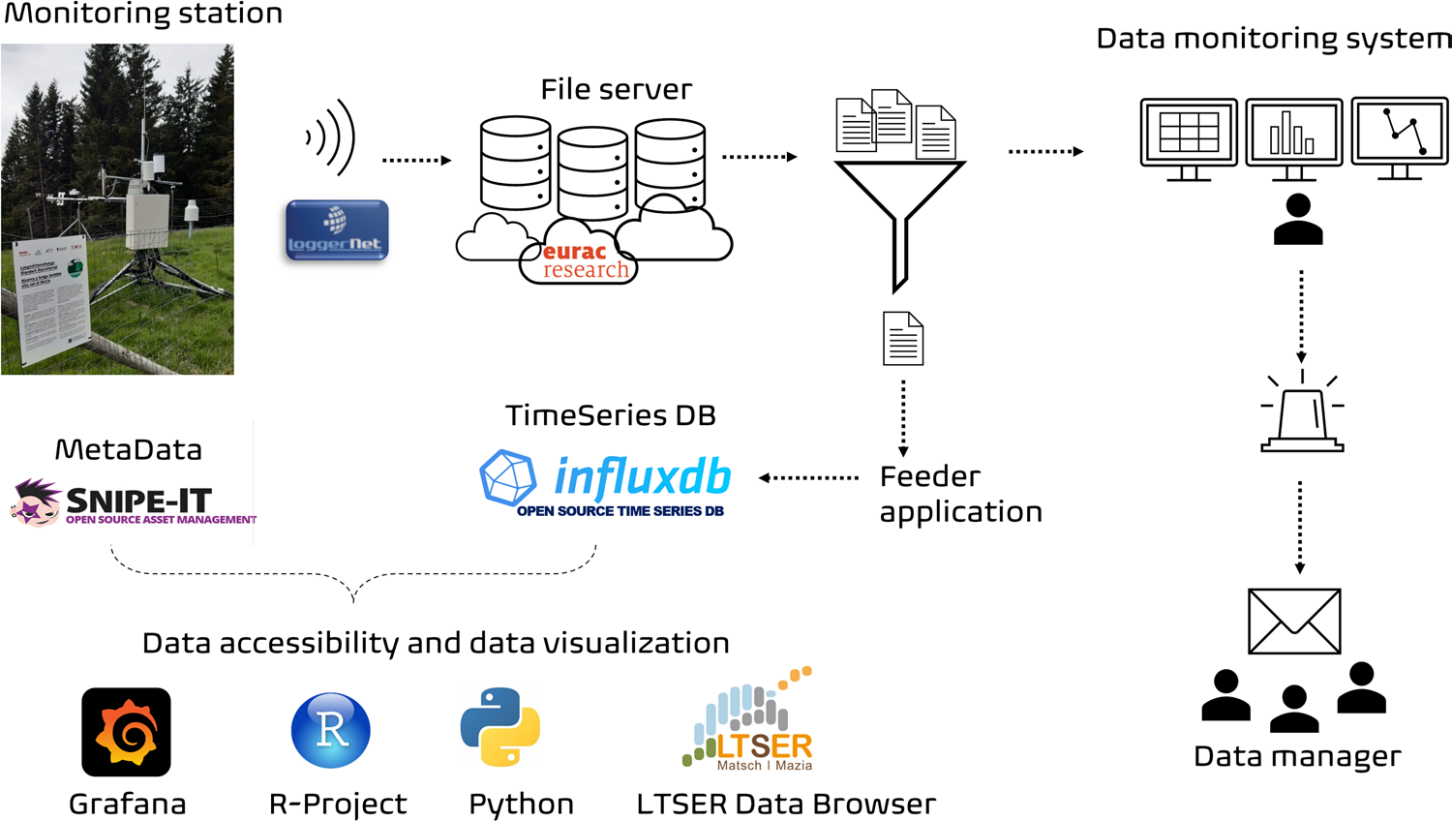
The workflow, developed in-house, starts from data acquisition at the monitoring station; then, the data are transmitted to the file server and the time series are stored in the database based on InfluxDB. An automatic data monitoring system is implemented, and the data are accessible to end users via several platforms.

The entire workflow, from data acquisition in the field to the transmission to our internal file server, database feeding, data download via web, and data visualisation using customised dashboards, is shown in Fig. 6 and further described by Palma *et al*.^40^. Integrated into the flow, a monitoring system performs two basic functions: checking the status of stations and sensors and alerting the system managers via email if there are malfunctions and performing preliminary data quality checks. The workflow consists of different, mainly open-source components and has been merged and integrated in-house.

All variables are stored locally in the measurement station logger memory; then, they are transferred to a file server on an hourly basis. Also on an hourly basis, these transferred data are checked and then stored in our time series database, which is based on InfluxDB (https://www.influxdata.com). At this point, the data are available for internal and external users.

Data accessibility is ensured in three different ways: i) Grafana (https://dashboard.alpenv.eurac.edu), an open-source application in which we have created a large number of dashboards that allow the data to be quickly visualized through graphs and allow the trends in the data to be analysed, where several measurements can be correlated in a single panel; ii) Mazia|Matsch Data Browser (https://browser.lter.eurac.edu^40^), a user-friendly web application developed in-house (with the Go language) that allows one to filter and download raw real-time data that have not been validated; and iii) in the Data Browser, there is a function that allows the direct generation of code templates in the R and Python languages, transforming the choices made by the users in the site menus into a query to download the data by running these scripts.

## Data Records

We produced one data file for each measured variable (15 in total) and two metadata files, which are stored and accessible in the Pangaea repository (10.1594/PANGAEA.964700^41^).

### Metadata files

The first metadata file is an Excel file that provides an overview of the measurement areas, the equipment of the stations, the sensor history, and the 15 recorded variables. The second metadata file is a pdf document that introduces the script used to perform quality control on raw data (DQC) and describes in detail the structure and content of the files generated by the script.

### Data files

For each of the 15 measurements, we provide a singular data file that includes raw data, as well as corrected and commented (elaborated) time series. The file structure and the headers used are in line with the standards of the Pangaea repository. Each data file consists of seven columns (Table 11), which provide the ‘Date/Time’ stamp (according to ISO 8601), the ‘Event’ indicating the name of the station, and the two geographic coordinates ‘latitude’ and ‘longitude’, which are followed by the raw value of the measurement (‘Variable abbreviation name [*unit*] (raw)’) and the elaborated variable value (‘Variable abbreviation name [*unit*]’). The first four headers are the same for all 15 data files, while the headers of columns 5 and 6 show the abbreviated name of the specific measurement, followed by the unit of measurement in square brackets and, in the case of column 5, the word ‘raw’ in round brackets to distinguish the original raw value from the elaborated value in column 6. The last column contains the tags set for the data quality check.

**Table 11:** Data file structure created for each variable after running the DQC script.

Column Nr.	Header	Header Description
1	Date/Time	Time series timestamp in UTC + 1 (format: YYYY-MM-DDThh:mm)
2	Event	Measurement station name
3	Latitude	Measurement station latitude
4	Longitude	Measurement station longitude
5	Variable abbreviation name [unit] (raw)	Raw time series (downloaded from https://browser.lter.eurac.edu^40^)
6	Variable abbreviation name [unit]	Elaborated time series (filtered with the DQC script)
7	Data_Quality	Qualifying data tag

A detailed description of the R script and the possible tags and associated actions that can be performed on the data can be found in the ‘Technical Validation’ section.

Missing data are indicated with ‘NA’ (Not Available) and can be caused by a data gap that is already present in the raw data and therefore reported in the elaborated data, or by the filtering performed by the DQC script, which may have detected inadmissible data for the reasons listed and described in detail in the ‘Technical Validation’ section.

Each data file contains the time series of processed data related to six years of measurements, for a total of over 210k values; the size of each file exceeds 50 MB, for a total dataset volume of almost 1 GB.

## Technical Validation

### Data quality check

The monitoring system (see Fig. 6), in addition to the alerting action, performs an automatic, basic check on an hourly basis on the data transmitted by the stations, creating the raw time series; it ensures the integrity of the data structure and the contents of the datasets. In the case of data overlaps or invalid character detection, it alerts the data manager, requesting a manual intervention.

To validate the datasets in more depth, a DQC script was developed that is able to process the raw time series. The DQC script is fed by a variable-specific instruction file that is created manually and reports anomalous events and the action to be taken in each case.

The instruction file contains the following columns: the ‘measurement station’, ‘start date’, and ‘end date’ of the event and the ‘event description’ and ‘value’ related to the event (the value is usually a threshold, an offset, etc.). The DQC script analyses each value of the raw time series and assigns a qualifying tag (associated with the instruction file) to each value; this is useful for any subsequent filtering. Depending on the tag assigned, the raw data undergo a transformation and, as a result, a new, elaborated time series is generated in the output.

### Data quality tags

The tags contained in column 7 of the data files are described, along with the associated filters, in Table 12.

**Table 12:** Description of tags set by the DQC script and filter action description.

Nr.	Data Quality	Data Quality Tag and Consequent Filter Action Description
1	Ok	Data passed the check successfully; no data manipulation
2	Data_gap	No value available; processed value = ‘NA’
3	Wrong	Data are corrupted and have been replaced with ‘NA’
4	Unreliable	Data are unreliable; no data manipulation
5	Lower_min	Data are lower than Threshold_min and have been replaced with ‘NA’
6	Upper_max	Data are greater than Threshold_max and have been replaced with ‘NA’
7	Lower_sub_min	Data are within the range [Threshold_min, Sub_threshold_min] (slightly below permissible values) and have been replaced with Sub_threshold_min value
8	Upper_sub_max	Data are within the range [Sub_threshold_max, Threshold_max] (slightly above permissible values) and have been replaced with Sub_threshold_max value
9	Unreliable/Lower_sub_min	Unreliable data are within the range [Threshold_min, Sub_threshold_min] and have been replaced with Sub_threshold_min value
10	Unreliable/Upper_sub_max	Unreliable data are within the range [Sub_threshold_max, Threshold_max] and have been replaced with Sub_threshold_max value
11	Offset	Data present a deviation from zero; ‘offset value’ has been added to the value
12	Irrigation	Data originated from artificial irrigation; no data manipulation

The tags ‘***Ok***’ and ‘***Data gap***’ do not require any external instructions, since the DQC script manages both automatically.

‘***Wrong***’ and ‘***Unreliable***’ tags are assigned to anomalous values present in the raw time series, adopting the time intervals detected and listed in the instruction file. Usually, these periods coincide with maintenance interventions or sensor failures. If data are definitely unrecoverable (e.g. in the case of data collection in the absence of the sensor), then these data will be tagged as ‘Wrong’ and the script will replace these data with ‘NA’ in the elaborated time series; otherwise, the data will be left unchanged but tagged with ‘unreliable’ to permit the future exclusion of these values.

The tags from rows 5 to 10 in Table 12 apply thresholds and sub-thresholds, which are necessary for the script to determine the action to apply to the raw data; Table 13 shows the list of thresholds used to eliminate or fix the outliers.

**Table 13:** Measurement thresholds set in the DQC filtering process.

Variable	Unit	Threshold_min	Threshold_max	Sub_threshold_min	Sub_threshold_max
TTT	°C	−27	35	/	/
RH	%	0	103	/	100
ff	m/s	/	30	/	/
dd	degrees	0	360	/	/
SWD	W/m^2^	−15	2000	0	/
Precip	mm	0	100	/	/
Snow h	m	−0.02	2.00 *	/	/
T soil	°C	/	/	/	/
vol SWC	m^3^/m^3^	0	1	/	/
Psi Soil	kPa	−10^5^	/	/	/

*B3 and S3 stations: threshold_max value exceptions:

B3

threshold_max = 1.25 m, except for the period between 10:45 and 13:00 on 2018-01-22.

S3

Threshold_max_1 = 2.78 m, applied from 00:00 on 2017-01-01 to 00:00 on 2019-09-18.

Threshold_max_2 = 2.20 m, applied from 00:15 on 2019-09-18 to 23:45 on 2022-12-31.

Whereas almost all thresholds in the table coincide with the operative limit of the sensors, in the case of the air temperature and wind speed, the thresholds have been restricted as much as possible around the extreme and, at the same time, admissible values recorded by the stations from the day of installation to the current day: this is to maximise the effectiveness of the outlier filter.

The ‘***Lower_min***’ and ‘***Upper_max***’ tags indicate that values that exceeded the respective thresholds have been replaced with ‘NA’. In some cases, it has been necessary to introduce sub-thresholds, which replace values that are only slightly outside of the norm, such as measurements of negative solar radiation during the night or relative humidity measurements that are above saturation.

Two specific tags that have been developed for the variables precipitation (tag ‘irrigation’) and snow height (tag ‘offset’) are briefly presented below.

### ‘Irrigation’ tag for precipitation

Two of the six measurement stations, B1 (983 m a.s.l.) and B2 (1473 m a.s.l.), collected precipitation data measured by rain gauges that were hit by the artificial irrigation of the surrounding meadow. Contrary to natural precipitation, irrigation occurs quite regularly, in terms of both the frequency and amplitude. Hence, we were able to identify each irrigation event by visually comparing both irrigated stations with the neighbouring stations, and then the instruction file was compiled with these irrigation periods.

### ‘Offset’ tag for snow height

The readings of ultrasonic distance sensors are highly influenced by changes in the station setup and by the vegetation growth below the station. This effect is especially pronounced in remote environments, where damage due to snow accumulation, wildlife interactions, and strong winds can significantly impact the station stability. In particular, displacements of the station structures and maintenance work might lead to sensor height changes. Consequently, our data quality check strategy incorporates specific offset adjustments, which are reported in our maintenance logbook for snow height measurements. After such deviations were manually detected, they were listed in the instruction file related to the snow height measurement.

During DQC, a specific sequence of steps was followed. Initially, values surpassing the sensor height were excluded (‘upper_max’ values), with one or two upper limits set for each station (Table 13). Subsequently, the eventual offset was applied to the raw snow height measurements.

The script applied either a constant offset or a dynamic offset, which is calculated in a linear increasing (or decreasing) manner, starting from the first value and going to the last value belonging to the affected period. After offset implementation, values lower than −0.02 m were removed (‘lower_min’). This threshold is comparable to the sensor’s measuring error of ±0.01 m. This step followed the application of the offset to prevent the unintentional removal of measurements falling below the threshold, considering that these values have not been adjusted for the offset at that point.

### Data completeness

**Fig. 7:**
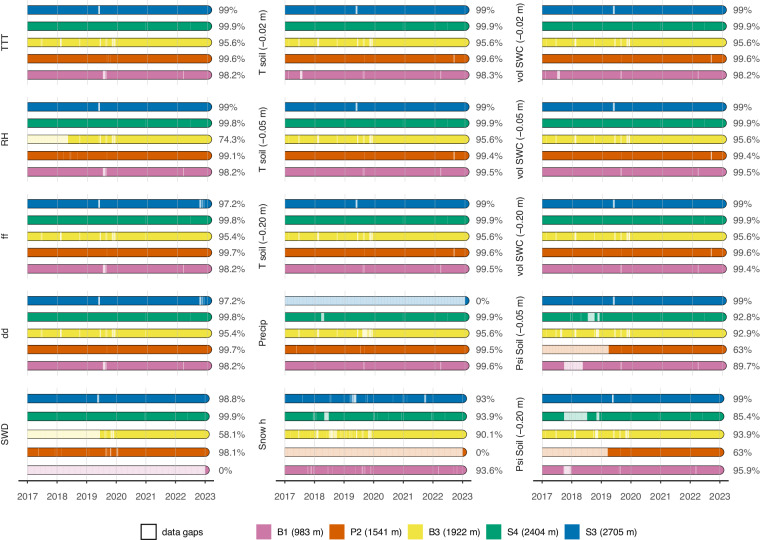
Comparison of data completeness for each of the 15 measurements for the five monitoring stations. The variables SWD and precipitation collected by the sixth station (B2) were included in the visualisation of station P2.

Some areas of the LTSER site Val Mazia – Matschertal are rather difficult to reach, and the two highest monitoring stations cannot be physically accessed throughout the whole year (i.e. the winter period), so that any failure in either the transmission system or PV system, or failures related to a sensor, can lead to data loss. Nevertheless, the total data gaps due to sensor failure over the entire six-year period make up 2.0% of the dataset. Additionally, data gaps related to the absence of a sensor for a certain period or for the whole period of the dataset make up 6.0% of the dataset, so that the overall completeness of the dataset is 92.0% (Fig. 7).

Thus, most of the missing data can be attributed to measurements that, at certain stations, were never performed or at least were not performed for long periods. For example, the measurement of short-wave radiation started in 2019 for station B3 (1922 m a.s.l.), and short-wave radiation measurements were never taken at station B1 (983 m a.s.l.). Precipitation was never measured at station S3 due to its elevation of 2705 m a.s.l. (requiring a heated rain gauge and consequently a robust power supply), and the snow height was not collected at P2 (1541 m a.s.l.). The collection of the soil water potential at 5 and 20 cm depth at P2 (1541 m a.s.l.) started in 2019.

Concerning anomalous measurements, the snow height is a sensitive variable, and data are often lost during normal sensor functioning. For example, during a snowfall event, the falling snowflakes will cross the beam of the ultrasonic device and interfere with the measurements; additionally, in the presence of vegetation, as the vegetation grows, the target becomes more and more inhomogeneous and difficult to measure. An algorithm has recently been added to the stations’ own data acquisition script that can reduce the data loss while providing more accurate measurements: instead of the last sample, the median value of the 15 samples is logged.

The air temperature and relative humidity datasets are in large parts complete for all five stations, even with the anomaly that affected some thermo-hygrometers, as explained in the ‘Methods’ section.

## Usage Notes

The meteorological time series presented in this paper can be accessed through Pangaea (10.1594/PANGAEA.964700^41^). The data are clean and can be used as is; gaps are not filled. In Pangea the data files of the 15 variables are aggregated in 8 timeseries: air temperature and relative humidity, wind speed and direction, solar radiation, soil temperature (at 2, 5, and 20 cm depth), soil water content (at 2, 5, and 20 cm depth), soil water potential (at 5 and 20 cm depth), precipitation, and snow height. Both timestamps are provided, UTC and UTC + 1 (local time).

The LTSER site Val Mazia – Matschertal runs other climate stations and collects additional measurements (raw data that have not been validated), which are freely available in near-real time (acknowledging our work) from our Data Browser (https://browser.lter.eurac.edu^40^) and can be visualised using a Grafana dashboard (https://dashboard.alpenv.eurac.edu).

## Data Availability

The codes, written in the R language and used for our monitoring system (https://gitlab.inf.unibz.it/alpenv/Station_Monitoring_System) (R version 3.6.0) and the DQC script (https://gitlab.inf.unibz.it/alpenv/ltser_datapaper) (R version 4.2.2), are freely available on the collaborative platform Gitlab. Furthermore, the DQC script is also provided with a DOI and persistently stored in Zenodo (https://zenodo.org/records/10255852).
